# Cross-cultural adaptation and psychometric validation of the Korean version of rehabilitation complexity scale for the measurement of complex rehabilitation needs

**DOI:** 10.1097/MD.0000000000026259

**Published:** 2021-06-18

**Authors:** Hoo Young Lee, Jung Hyun Park, Tae-Woo Kim

**Affiliations:** a TBI Rehabilitation Center, National Traffic Injury Rehabilitation Hospital, Gyeonggi-do; b Department of Rehabilitation Medicine, Seoul National University Hospital, Seoul University College of Medicine, Seoul; c Traffic Injury Rehabilitation Research Institute, National Traffic Injury Rehabilitation Hospital, Gyeonggi-do; d Department of Medicine, Yonsei University College of Medicine, Seoul; e Department of Rehabilitation Medicine, Gangnam Severance Hospital, Rehabilitation Institute of Neuromuscular Disease, Yonsei University College of Medicine, Seoul, South Korea.

**Keywords:** disability, factor analysis, measures, outcomes, rehabilitation, therapy, validation study

## Abstract

The aim of this study was to translate and cross-culturally adapt the Rehabilitation Complexity Scale-Extended version 13 (RCS-E v13) to develop the Korean version of the Rehabilitation Complexity Scale (KRCS), and to explore its reliability, and concurrent and construct validity.

This research was an observational study of a series of consecutive rehabilitation inpatients who were previously assessed with KRCS and grouped with the Korean rehabilitation patient group version 1.1 (KRPG v1.1). Translation and cross-cultural adaptation of the RCS-E v13 were implemented according to internationally recognized standards. Four hundred thirty inpatients diagnosed with complex neurological or musculoskeletal disabilities were enrolled. Physiatrists were asked to finish the KRCS at admission and to complete a second time with an interval of a minimum of 3 weeks to a maximum of 4 weeks for reliability evaluation. At discharge, the KRCS was completed a third time to explore constructive validity.

The Cronbach-α was 0.63. The intraclass correlation coefficient values of the total score, Medical, Nursing, Care, Therapy Disciplines, Therapy Intensity, and Especial Needs domains were 0.86, 0.69, 0.84, 0.83, 0.74, 0.74, and 0.79, respectively (*P* < .01). The scale was repeatable (Spearman rho 0.69–0.86) and correlated strongly with disability measures (Spearman rho 0.37–0.50). Exploratory factor analysis revealed 2 clear factors (“Medical/Nursing” and “Care/Therapy Disciplines/Therapy Intensity/Equipment”). The goodness-of-fit index in the confirmatory factor analysis was 0.87. The KRCS was associated with a higher explanatory power for rehabilitation resources and length of stay than the KRPG v1.1.

Our data suggest that the KRCS is a feasible, reliable, and valid tool that is appropriate for the measurement of clinical complexity in Korean intensive rehabilitation units. Further, it may provide case-mix adjustment to improve the rehabilitation delivery system in Korea.

## 1 Introduction

Assessment of the complexity of rehabilitation needs is important in rehabilitation medicine in terms of quality of care, patient flow, and resource allocation.^[1,2]^ Standardized measurement of rehabilitation complexity is a worldwide challenge.^[3,4]^ Moreover, a wide variation in cost is observed in complex areas of rehabilitation medical services.^[5,6]^

In Korea, a 3-stage rehabilitation care delivery system is being considered. Acute rehabilitation is provided at general and tertiary hospitals, subacute rehabilitation is provided at inpatient rehabilitation institutions, and chronic rehabilitation is provided by outpatient or long-term rehabilitation services. However, there is no criteria or regulation for inpatient rehabilitation across the delivery system.^[7]^ The rehabilitation institution makes a voluntary decision on the length of hospitalization and the number of rehabilitation services. Thus, a rehabilitation medical institution system was recently established in Korea to guarantee hospitalization period and rehabilitation services for subacute patients who need intensive rehabilitation services according to the timing and circumstances of the onset of a condition.

The Korean rehabilitation patient group version 1.1 (KRPG v1.1) is currently utilized to reflect the characteristics of rehabilitation inpatients as the case-mix and payment tool. The etiologic disease, functional status (cognitive function, activity of daily living, muscle strength, spasticity, level, and grade of spinal cord injury), and the patient's age are the variables in the patients with brain or spinal cord injury. Patients with musculoskeletal problems or amputation are classified only by age.

Previous study elucidated that the first version of the Korean rehabilitation patient group explained just 11.8% of the variance in charge for rehabilitation inpatients. In the most recent study, KRPG v1.1 explained 8.6% of the variance in charge for rehabilitation inpatients with acquired brain injury. Lack of accuracy and comprehensiveness of the KRPG v1.1 as the case-mix and payment tool for rehabilitation inpatients have been problematic issues.^[8]^

The Rehabilitation Complexity Scale (RCS) was introduced in 2007 to evaluate the complexity of rehabilitation needs. The RCS incorporates patient's real rehabilitation needs and it is practical and feasible for implementation in clinical practice.^[5,9]^ The RCS-Extended version 13 (RCS-E v13) is currently utilized to measure the rehabilitation needs of patients in the UK Rehabilitation Outcome Collaborative Database. Recent studies demonstrated positive cross-cultural validation of the Danish and the Italian RCS-E v13.^[10–13]^

To establish a rehabilitation medical delivery system under the Korean circumstances, the current KRPG v1.1 alone is insufficient to address the complex rehabilitation needs because it solely depends on diagnostic, age-related, and disability measures. Therefore, it is necessary to complement the KRPG v1.1 and develop a more accurate, comprehensive, and feasible evaluation system based on the complexity of the needs as well as diagnostic and disability measures.

The aim of this study was to translate and cross-culturally adapt the RCS-E v13 to generate the Korean version of the RCS (KRCS), to explore its reliability, and concurrent and construct validity, and to demonstrate its complementary value of KRCS by comparing KRPG v1.1 with the KRCS and the KRPG combined in identifying the rehabilitation needs and resources for patients hospitalized in rehabilitation institutions.

## 2 Methods

### 2.1 Participants and setting

This research is a retrospective observational study and included data from a total of 430 inpatients (234 males and 196 females) with neurological injury occurring within 3 months of onset or surgery, hip replacement after femur or hip fracture within 1 month of onset or surgery, or lower extremity amputation performed within 2 months of onset or surgery who admitted to 6 rehabilitation medical institutions and agreed on the subacute intensive rehabilitation program from January to August 2018. A sub-sample of 199 patients was also collected at discharge for the confirmatory factor analysis (CFA). A 24-hour medical care is provided in the rehabilitation medical institution for patients who cannot be treated as outpatients.

### 2.2 Korean rehabilitation patient group version 1.1

The KRPG v1.1 consists of different variables according to the disease group. The variables in the acquired brain injury group include age, Korean Version of Mini-Mental Status Examination, the Korean version of the Modified Barthel Index, Manual Muscle Testing, and Modified Ashworth Scale. Variables of spinal cord injury group include age, Manual Muscle Testing, Spinal Cord Independence Measure, the combination of the neurological level of injury and American Spinal Injury Association Impairment Scale, and Modified Ashworth Scale. Variables in the musculoskeletal injury group include age, the Korean version of the Modified Barthel Index, and Manual Muscle Testing.

### 2.3 Translation and cross-cultural adaptation

With the permission of the developer of RCS-E v13, translation and cross-cultural adaptation of the RCS-E v13 were implemented according to internationally accepted and recommended guidelines to develop the KRCS.^[14,15]^ A medical doctor fluent in English and an interpreter who had lived in an English-speaking country independently translated the RCS-E v13 into the Korean language. After the reconciliation of these 2 forward translations into a single forward translation, 2 distinct interpreters whose first language is English and who are neither aware nor informed of the scale translated the last forward translation back into the original language (English). The final consensus exercise was done according to the medical conditions in Korea by adapting the modified Delphi survey method to achieve a consensus with 16 experts in rehabilitation.

### 2.4 Procedures of the modified Delphi

Two Delphi surveys were conducted through email from March to April 2018 to confirm the content validity of the initial KRCS. The panel of 16 experts in this research consisted of executives of the Korean Academy of Rehabilitation Medicine, the Korean Medical Association, and the Korean Physiatrists Association Korean Academy of Rehabilitation Medicine and physiatrists who provide rehabilitation services at the general and tertiary hospitals and inpatient rehabilitation institutions who are able to present professional opinions regarding the rehabilitation patient grouping system and the rehabilitation medical delivery system, and executives of the Korean Academic Society of Rehabilitation Nursing who were also experts with a position above a nursing director at the general and tertiary hospitals, or a position above that of academic staff at a nursing college.

The first Delphi survey was based on the initial KRCS items to address appropriateness in terms of cross-cultural adaptation. Each domain and item was evaluated by a 4-point Likert scale (1 point: not at all appropriate, 2 points: not appropriate, 3 points: appropriate, and 4 points: very appropriate). “Appropriateness” referred to the level of significance of the items in measuring the complexity of needs and suggesting a direction of the rehabilitation services.

The second Delphi survey was conducted based on the results of the first Delphi survey to inform the panel of the indicators adopted, revised/supplemented, and deleted for their reference in reevaluation. The participants were asked to describe freely their opinions on each item. As with the first Delphi survey, the “appropriateness” of each item was evaluated on a 4-point Likert scale.

The Content Validity Index (CVI) was calculated for each survey stage to verify the content validity of each item. A CVI value of 0.8 or greater was considered appropriate.^[16–18]^ Those with 0.5 to less than 0.8 were discussed (mediated differences of opinions), and those with less than 0.5 were dropped. The final version was established after implementing all the necessary adjustments.

### 2.5 Reliability

Internal consistency of the KRCS was examined using Cronbach alpha with 95% confidence intervals. Values of 0.6–0.7 were considered acceptable and value ≥0.7 were regarded as satisfactory.^[19]^

Reproducibility of test–retest repeatability was evaluated for individual items and total scores using the intra-class correlation coefficient (ICC). The interval between the test and the retest was limited from a minimum of 3 weeks to a maximum of 4 weeks to preclude the possibility of carry-over effects between the tests.

### 2.6 Criterion validity

Spearman rho correlations were used to test criterion validity between the KRCS and other standards (eg the Korean Rehabilitation Patient Group Version 1.1, Korean Version of Mini Mental Status Examination, the Korean version of the Modified Barthel Index, Spinal Cord Independence Measure, the combination of the neurological level of injury and American Spinal Injury Association Impairment Scale, Manual Muscle Testing, and Modified Ashworth scale).

### 2.7 Construct validity

Construct validity was assessed using both exploratory and CFA. First, the Kaiser–Mayer–Olkin (KMO) measure and Bartlett test of sphericity were assessed to confirm the validity of factor analysis.

Exploratory factor analyses involving principal component analysis with orthogonal (Varimax) rotation were conducted to evaluate the dimensionality of the scale.^[20]^ The inter-factor correlation was studied. CFA was conducted using the data collected from 199 patients at discharge, where scores were distinct from data used for exploratory factor analysis, to use a cross-validation design.

### 2.8 Explanatory power

We investigated the coefficient of determination or the reduction of variance or R-squared of the KRCS with respect to the total cost, rehabilitation cost, medical cost, and length of stay and compared it with the KRPG version 1.1. R-squared explains how much variance of the data is “explained” by the model. “To explain” means to reduce the residual variance. Thus, the coefficient of determination is the ratio of explained variance to the total variance that tells about the strength of association between the variables.

$$R^{2}=\frac{\Sigma \text{i}(yi-A)2-\Sigma \text{i}(yi-Ag)2}{\Sigma \text{i}(yi-A)2}$$

*yi*: medical expenses for the ith patient

*A*: Overall average value of medical expenses

*A*_*g*_: Average value of medical expenses in the *g* group

### 2.9 Ethical considerations

This study was approved by the Institutional Review Board of National Traffic Injury Rehabilitation Hospital (No. NTRH-18003). It was conducted in accordance with the Declaration of Helsinki.

### 2.10 Statistical analysis

For all tests, a level of significance of *P* < .05 was used. The analysis was performed using SAS version 9.4 (SAS Institute, Cary, NC) and R software (version 3.5.2; The Comprehensive R Archive Network: http://cran.r-project.org), using traditional methods within the outline recommended by the COSMIN taxonomy.^[21]^

## 3 Results

### 3.1 The KRCS

In Korea, all of the services within the scope of hospitalization and rehabilitation are based on the doctor's prescription, hence, costs of medical care and rehabilitation services are subject to the doctor's prescription. Consequently, the prescription for patient assessment, medical care, and rehabilitation services is entirely a doctor's action. For example, in Korea, evaluation of the medical, nursing, and therapy needs are done by physiatrists whereas each need in original RCS-E v13 are evaluated by each discipline (eg, medicine, nursing, and therapy). Therefore, a cross-cultural adaptation of RCS-E v13 was needed to be carried out.

The KRCS is a 26-point measure comprising 5 domains: Medical (0–5 points); Nursing (0–5 points); Care (0–4 points); Therapy (including the Therapy Disciplines and the Therapy Intensity. Therapy Disciplines + Therapy Intensity: 0–9 points); and Especial Needs (0–3 points), whereas the RCS-E v13 includes 5 domains: Care or Risk (0–4 points), Nursing (0–4 points), Therapy (0–8 points), Medical (0–4 points), and Equipment Needs (0–2points) and the total score is set at a maximum of 22 points. The higher the score, the more complex the needs are. Since the risk domain of RCS-E v13 concerns intellectual or behavioral disability, this domain was uninvolved in the KRCS according to the conditions and inpatient composition of the rehabilitation medical institutions in Korea.

“Medical” refers to the medical needs of rehabilitation patients and consists of 6 items. It mainly represents the degree of medical care needed by the patients. It may distinguish patients who need medical care at tertiary hospitals, general hospitals (number of beds is more than or equal to 100), rehabilitation medical institutions, or outpatient clinics. “Nursing” denotes nursing needs including 6 items. In case of no need for skilled nursing, it is classified as Nursing 0, whereas the need for qualified nursing during hospitalization is indicated as Nursing 1–3, and the need for highly special nursing as Nursing 4 and Nursing 5. “Care” corresponds to “delegable nursing or caring needs.” In Korea, classifying the needs for care depending on the amount of time or number of caregivers may cause conceptual misunderstanding between care and compensation. Therefore, the “Care” domain in the KRCS refers to the requisite level of partial or continuous care. “Therapy” denotes rehabilitation therapy needs and is most similar to the RCS-E v13 in the United Kingdom. Items in “Therapy Disciplines” were modified to reflect the rehabilitation therapy in Korea. In terms of “Therapy Intensity,” 4 and 5 correspond to intensive rehabilitation during acute and subacute phases, whereas 0, 1, 2, and 3 correspond to rehabilitation in the chronic phase. “Especial Needs” refers to “other professional rehabilitation medical needs.” It is the most modified content in the RCS-E v13 because it reflects the clinical scenario and delivery system of rehabilitation medicine in Korea. It refers to the need for professional elements of rehabilitative medicine, which may be provided at a limited number of rehabilitation institutions or tertiary hospitals. No need for professional rehabilitation is classified as 0, the need for the referral at the regional level for specialized examination or therapy is classified as 1, and the referral need across the regional level is classified as 2.

### 3.2 Demographic characteristics and descriptive analysis of the scale

Participant characteristics are presented in Table 1. Of the 430 participants, 365 (84.9%) had acquired brain injury and 29 (6.7%) had spinal cord injury; 234 (54.4%) were male and 196 (45.6%) were female. The age ranged from 20 to 96 years with a mean age of 67 [SD: 14 years] years. The median length of stay was 50 days.

**Table 1:** Demographic characteristics of the study population (n = 430).

Age, years, mean, (SD), min-max	67 (14), 20–96
Sex, women/men, n	196/234
Length of stay, median, (IQR)	50 (26–108.5)

Korean Rehabilitation Impairment Category		Number	%
1	Cerebrovascular accident	332	77.21
2	Traumatic brain injury	25	5.81
3	Other acquired brain injury	8	1.86
5	Traumatic spinal cord injury	10	2.33
6	Other spinal cord injury	19	4.42
7	Brain and spinal cord injury	2	0.47
12	Hip fracture	30	6.98
13	Lower limb arthroplasty	4	0.93
14	Lower limb amputation	0	0

Total scores on the KRCS ranged from 0 to 26. The median values for the total score, Medical, Nursing, Care, Therapy Disciplines, Therapy Intensity, and Especial Needs domains were 16, 2, 2, 3, 3, 5, and 1, respectively.

### 3.3 Content validity

The CVI values of items in the KRCS were 0.86–1.0 and S-CVI/Ave was 0.93, respectively. Therefore, none of the items was excluded during psychometric evaluation.

### 3.4 Reliability

The Cronbach alpha of the KRCS was 0.63 and there was no single item that if deleted would improve the overall Cronbach alpha value in the scale. The test–retest reliability was examined using the ICC. The ICC values of the total score, Medical, Nursing, Care, Therapy Disciplines, Therapy Intensity, and Especial Needs domains were 0.86, 0.69, 0.84, 0.83, 0.74, 0.74, and 0.79 (*P* < .01).

### 3.5 Criterion validity

The results of the validity and relationship of the KRCS with the other measures are presented in Table 2. Spearman correlation coefficient showed a significant correlation between the total score, Nursing, Care domains and Korean Version of Mini Mental Status Examination, the Korean version of the Modified Barthel Index and Manual Muscle Testing in patients with acquired brain injury (*ρ* = 0.37–0.50) while Therapy Disciplines, Therapy Intensity, and Especial Needs domains showed a weak correlation.

**Table 2:** Spearman rank correlations between the Korean version of the Rehabilitation Complexity Scale and the Korean Rehabilitation Patient Group version 1.1.

	Medical	Nursing	Care	Therapy Disciplines	Therapy Intensity	Especial Needs	Total score
Korean Rehabilitation Impairment Category 01,02,03							
Age	0.14	0.29	0.24	0.15	0.19	−0.08	0.24
Korean Version of Mini Mental Status Examination	−0.17	−0.46^†^	−0.40^†^	−0.26	−0.16	−0.03	−0.44^†^
Korean version of the Modified Barthel Index	−0.27	−0.49^†^	−0.50^†^	−0.28	−0.30	−0.10	−0.53^†^
Manual Muscle Testing	−0.24	−0.37^†^	−0.40^†^	−0.22	−0.25	−0.07	−0.41^†^
Modified Ashworth Scale	0.03	−0.04	−0.04	−0.03	−0.11	0.09	−0.05
Korean Rehabilitation Impairment Category 05,06							
Age	0.17	0.05	0.37^∗^	0.13	0.24	−0.33	0.07
Manual Muscle Testing	−0.63^†^	−0.53^†^	−0.53^†^	−0.14	−0.19	0.37	−-0.50^†^
Modified Ashworth Scale	−0.46^∗^	−0.12	−0.17	−0.38^∗^	−0.16	0.23	−0.13
Spinal Cord Independence Measure	−0.46^∗^	−0.51^†^	−0.69^†^	−0.31	−0.29	0.23	−0.59^†^
Combination of neurological level of injury and American Spinal Injury Association Impairment Scale	−0.49^∗^	−0.42^∗^	−0.35	−0.31	−0.40^∗^	0.17	−0.41^∗^
Korean Rehabilitation Impairment Category 12,13							
Age	−0.24	−0.10	−0.05	0.24	−0.18	−0.13	−0.10
Modified Barthel Index	−0.29	−0.18	−0.30	−0.32	0.27	−0.31	−0.27
Manual Muscle Testing	−0.25	−0.09	−0.22	−0.34	0.29	−0.36^∗^	−0.37^∗^

### 3.6 Construct validity

Before the exploratory factor analyses, KMO test and Bartlett test of sphericity were conducted to evaluate the factorability. The KMO value was 0.64, and the value of Bartlett test of sphericity was 340.96, with statistical significance (*P* < .001).

**Figure 1:**
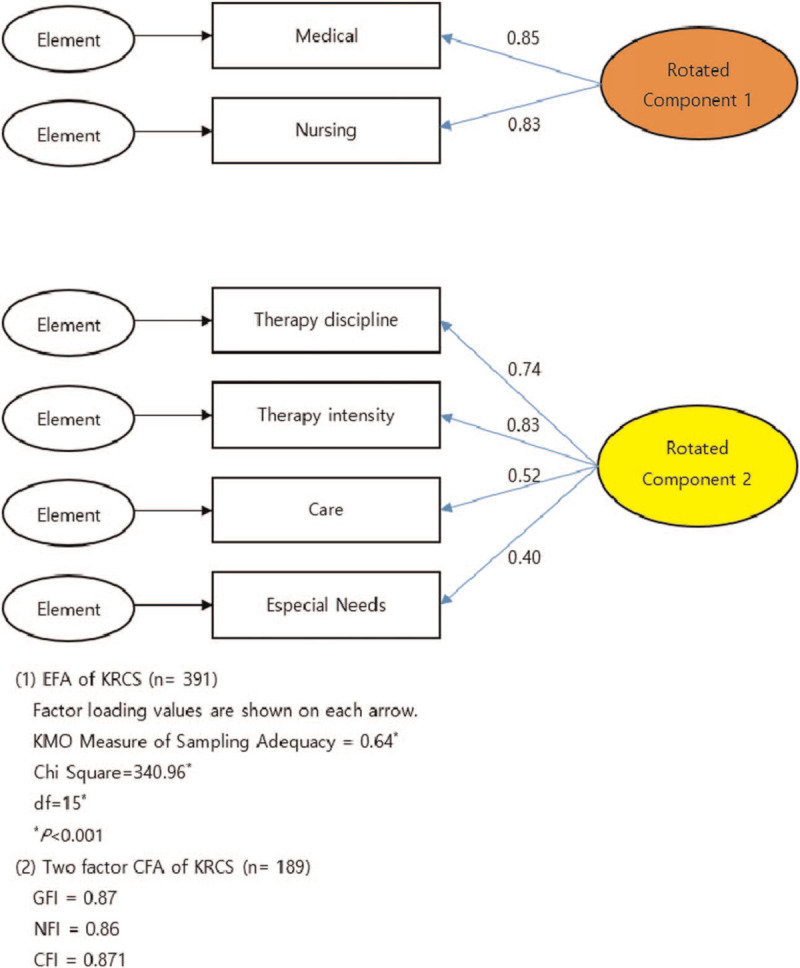
Results of exploratory factor analysis of the KRCS scores at admission (n = 391) and two-factor confirmatory analysis at discharge (n = 189). CFA = confirmatory factor analysis, CFI = comparative fit index, df = degree of freedom, EFA = exploratory factor analysis, GFI = goodness-of-fit-index, KMO = Kaiser–Meyer–Olkin, KRCS = the Korean version of Rehabilitation Complexity Scale, NFI = normed fit index, RC = rotated component, Sig = significance, SRMR = standardized root mean square residual.

Table 3 and the path diagram in Figure 1 display the results of the exploratory factor analyses, which were conducted using principal component analysis and Varimax orthogonal rotation by R software (version 3.5.2). Only the first 2 components had an eigenvalue >1, together accounting for 56.63% of the total variance in scores. All 6 domains of the KRCS administered to patients were “moderate” to “high” on the first unrotated principal component with loadings ranging from 0.46 to 0.71. Parallel analysis indicated a 2-factor solution, which was rotated using a Varimax procedure: the first factor appeared to be “Medical/Nursing,” which accounted for 35.91% of the variance. The second factor appeared to be “Care/Therapy Disciplines/Therapy Intensity/Especial Needs,” accounting for 20.72% of the variance. Convergent and discriminant validities for all constructs and the known group validity for 2 constructs were established.

**Table 3:** Results of exploratory factor analysis of domains in the Korean version of the Rehabilitation Complexity Scale.

	Unrotated principal component loading		Varimax rotation orthogonal factor loading	
Domains	Principal component 1	Principal component 2	Rotated component 1	Rotated component 2
Medical	0.57	−0.63	0.85	−0.05
Nursing	0.71	−0.46	0.83	0.17
Care	0.69	0.05	0.45	0.52
Therapy Disciplines	0.46	0.59	−0.09	0.74
Therapy Intensity	0.65	0.53	0.09	0.83
Especial Needs	0.48	0.09	0.28	0.40

The model fit indices were as follows: goodness-of-fit-index = 0.87, Bentler–Bonett normed fit index = 0.86, comparative fit index = 0.87, and standardized root mean square residual = 0.09.

### 3.7 Explanatory power

The KRCS showed higher explanatory power than the KRPG v1.1. The R-squared values of the KRCS were 0.14, 0.13, 0.20, and 0.38, while the R-squared values of the KRPG v1.1 were 0.12, 0.12, 0.14, and 0.34 for the total cost, rehabilitation cost, medical cost, and length of stay, respectively. After merging the Korean version of the KRCS with the KRPG v1.1, the coefficients of determination increased to 0.29, 0.38, 0.44, and 0.51 for the total cost, rehabilitation cost, medical cost, and length of stay, respectively.

## 4 Discussion

In this study, we developed the KRCS via translation and cross-cultural adaption of the RCS-E v13. We also verified the reliability and psychometric validity based on a sample of 430 patients in the intensive and comprehensive rehabilitation phase with highly complex rehabilitation needs in Korea. The KRCS results revealed adequate feasibility, reliability, and concurrent validity. A two-factor solution involving “Medical/Nursing” and “Care/Therapy Disciplines/Therapy Intensity/Especial Needs” was demonstrated while the model fit indices were slightly less than the good fit values. The KRCS showed higher explanatory power than the KRPG v1.1 in terms of service cost and length of stay. After merging the total score of the KRCS into the Korean Rehabilitation Patient Group Version 1.1, the explanatory power was more than twice as high as the explanatory power of the Korean Rehabilitation Patient Group Version 1.1. This is the first study to develop and verify the RCS-E v13 that may complement the explanatory power of the Korean Rehabilitation Patient Group Version 1.1, which is currently used to classify patients and services in the rehabilitation delivery system of Korea.

The structure of the RCS specifies the patient's real needs and appropriate rehabilitation setting. It facilitates individual identification of the medical, nursing, care, therapeutic, and other rehabilitation needs, as well as the overall complexity of the rehabilitation domain.^[5,9,22]^ Furthermore, the assessment of “Especial Needs” in the KRCS, or “other professional rehabilitation medical needs” is based on the description of the patient's condition from a non-medical perspective. Vocational, driving, or sexual rehabilitation needs, which need referral across the regional level in Korea, are classified as E2. Hence, the KRCS is based on the World Health Organization guidelines for rehabilitation, which emphasize maximization of recovery and maintenance of optimal levels of independence and functioning.^[20]^

Furthermore, the KRCS appears suitable for a case-mix adjustment and payment tool to harmonize the rehabilitation outcome and service cost, especially for highly complex cases.^[23]^ The KRCS outperformed the KRPG v1.1 in predicting the service cost and length of hospitalization. The KRPG v1.1 explained 11.8% of the variance in rehabilitation charges for patients with brain or spinal cord injury in the subacute phase.^[8]^ It also accounted for 13.8% of the variance in the length of stay. The explanatory power of the KRPG v1.1 in our study yielded similar results in that the KRPG v1.1 explained 11.5% of the variance in the total service cost. However, it explained 34.4% of the variance in the length of stay. Merging of the total score of KRCS into the KRPG v1.1 increased the explained variance in the total cost, rehabilitation cost, and medical cost to more than twice as high as that of KRPG v1.1 or KRCS alone. Therefore, by complementing KRPG v1.1, KRCS enabled the determination of the cost of rehabilitation programs delivered to provide cost-effective rehabilitation services. Similarly, a previous study, which measured acute rehabilitation needs in patients with traumatic injuries, showed that the RCS outperformed the injury severity score and the Barthel index by explaining the rehabilitation needs associated with injury severity, rehabilitation complexity, length of stay, and discharge destination.^[24]^

While the KRCS requires the comprehensive and professional expertise of rehabilitation medicine physicians at the time of evaluation, the outcome is intuitive and simple. Hence, anyone associated with health care delivery (ie, nurses, therapists, insurance reviewers, and social workers) can easily understand and utilize the tool.

Some limitations of the present study need to be taken into account during the interpretation of results. First, the initial scores of patients who were already hospitalized at the start of the study might not be completely consistent with their actual rehabilitation needs at admission. Second, the sample size was not inadequate since KMO was 0.64, but a larger sample size might strengthen data integrity.^[25]^ Thirdly, this study was conducted at only a single level of the delivery system of rehabilitation medicine in Korea, that is, the intensive rehabilitation hospitals in the subacute phase. Larger and longitudinal studies are necessary for the precise evaluation of reliability and validity. Moreover, measurement of patients’ rehabilitation needs using the KRCS in the whole spectrum of rehabilitation medical service delivery system is required to investigate the gaps between needs and services and to establish the appropriate service delivery model in Korea.^[26]^

Taken together, we conclude that the KRCS is a feasible, reliable, and valid tool that is appropriate for the measurement of clinical complexity in Korean intensive rehabilitation units. Further, it provides a measure based on case-mix adjustment to determine the rehabilitation setting and resources allocated to an individual patient within the rehabilitation process as a gatekeeping tool in the rehabilitation delivery system of Korea.

## Acknowledgments

This study was supported by the R&D grant (No. 5-2018-A0024-00001) for rehabilitation by Korea National Rehabilitation Center Research Institute, Ministry of Health & Welfare. Special thanks are due to Professor Lynne Turner-Stokes, the author of the scale, who provided the RCS-E v13, as used today in the United Kingdom, and assisted us with valuable comments on the development of the KRCS. Further thanks are due to Hyung-Ik Shin, Seong Hoon Lim, Jeehyun Yoo, Sun Im, Myung Eun Chung, Soon Yong Kwon, Hyun-Mi Oh, and Jihye Park who provided expert opinion on the development of the KRCS and further utilization of the scale for the establishment of rehabilitation medicine delivery system of South Korea. Moreover, the Medical Research Collaborating Center, Seoul National University Hospital, Seoul University College of Medicine, Seoul, South Korea provided statistical assistance.

## Author contributions

**Conceptualization:** Hoo Young Lee, Tae-Woo Kim.

**Data curation:** Hoo Young Lee.

**Formal analysis:** Hoo Young Lee.

**Funding acquisition:** Tae-Woo Kim.

**Investigation:** Hoo Young Lee, Jung Hyun Park.

**Methodology:** Hoo Young Lee, Jung Hyun Park, Tae-Woo Kim.

**Project administration:** Tae-Woo Kim.

**Resources:** Hoo Young Lee.

**Software:** Hoo Young Lee.

**Supervision:** Jung Hyun Park, Tae-Woo Kim.

**Validation:** Hoo Young Lee, Tae-Woo Kim.

**Visualization:** Hoo Young Lee.

**Writing – original draft:** Hoo Young Lee, Jung Hyun Park.

**Writing – review & editing:** Hoo Young Lee, Tae-Woo Kim.
